# Atypical antipsychotic prescription for agitation in care homes: A qualitative study exploring views of residents, relatives and health and social care professionals

**DOI:** 10.1002/alz70857_104461

**Published:** 2025-12-25

**Authors:** Byron Creese, Douglas Macfarlane, Clive G Ballard, Nalamotse Joshua Choma, Joash Ding, William E Henley, John Dennis, Christoph Mueller, Nefyn Williams, Alys Griffiths, Ikran Dahir

**Affiliations:** ^1^ Brunel University of London, London, United Kingdom; ^2^ University of Exeter, Exeter, United Kingdom; ^3^ University of Exeter, Exeter, Devon, United Kingdom; ^4^ King's College London, London, United Kingdom; ^5^ University of Liverpool, Liverpool, United Kingdom; ^6^ University of Sheffield, Sheffield, South Yorkshire, United Kingdom

## Abstract

Atypical antipsychotics are either unlicensed or licensed with restrictions on duration and indication in many jurisdictions including USA, Canada, Europe and Australia. Despite this, prescribing data suggest regular use off label but the reasons for this are unclear. Exploring these reasons is central to managing safer prescribing and the most appropriate way to do this is via a detailed examination of the context in which prescribing occurs, including factors affecting initiation and maintenance.

This qualitative study aimed to understand patient, carer and clinician perceptions around the risks and benefits of atypical antipsychotics and preferences on how side effect risks are communicated. Semi‐structured interviews were conducted with care home residents, relatives, and staff. A focus group was conducted with external healthcare professionals. Using framework analysis, five main themes were identified: 1) the interplay between the person and their symptoms; 2) balancing voices in decision making; 3) what happens before prescription?; 4) bringing together information to make the right decision; 5) what happens after prescription?

Care home staff and relatives of people with dementia were generally uncertain of the risks of antipsychotics, and the potential side effects were often not explained adequately. Person‐centred care was preferred by participants. While antipsychotics continue to be prescribed, more efforts to minimise harm are required. Specifically, improved education around the risks of antipsychotic use in dementia is needed. Relatives wish to be involved in the decision‐making process alongside care staff, and their involvement should be optimised with the use of decision‐aids. Accessible dementia‐specific guidance on monitoring antipsychotics after prescription should also be developed. Finally, qualitative research taking the views of relatives, carers and clinicians should form a central part of guidance development for pharmacotherapy in dementia.

